# Draft Genome Sequences of Six Moroccan Helicobacter pylori Isolates Belonging to the hspWAfrica Group

**DOI:** 10.1128/MRA.00714-20

**Published:** 2020-10-08

**Authors:** Souad Kartti, Najat Bouihat, Nargisse El Hajjami, Mouna Ouadghiri, Tarik Aanniz, Mostafa Elouennass, Lahcen Belyamani, Azeddine Ibrahimi, Amina Benaouda

**Affiliations:** a Biotechnology Laboratory (MedBiotech), Bioinova Research Center, Rabat Medical and Pharmacy School, Mohammed V University, Rabat, Morocco; b Microbiology Laboratory, Cheick-Zaid University Hospital, Abulcasis University of Health Sciences, Rabat, Morocco; c Bacteriology Laboratory, Military Instruction Hospital Mohammed V, Bioinova Research Center, Rabat Medical and Pharmacy School, Mohammed V University, Rabat, Morocco; d Emergency Department, Military Instruction Hospital Mohammed V, Bioinova Research Center, Rabat Medical and Pharmacy School, Mohammed V University, Rabat, Morocco; Loyola University Chicago

## Abstract

Helicobacter pylori affects up to 50% of people worldwide. Here, we present the draft genome sequences of six H. pylori strains isolated from Moroccan patients with different gastric diseases. Multilocus sequence typing analysis showed that all of the H. pylori isolates belonged to the hspWAfrica group.

## ANNOUNCEMENT

Helicobacter pylori is a Gram-negative bacterium with four to six flagella. This human gastric pathogen colonizes almost 50% of the world’s population (1). H. pylori is a major etiological agent for a wide range of gastric diseases, such as gastritis, peptic ulcers, gastric carcinoma, and mucosa-associated lymphoid tissue lymphoma (2). In Morocco, the most common pathology resulting from H. pylori infection is chronic gastritis, with a rate of 66%, and the risk of developing gastric cancer among infected patients in this population is about 9% (3). Earlier studies on the structure of H. pylori populations using multilocus sequence typing (MLST) based on seven standard housekeeping genes (*atpA*, *efp*, *mutY*, *ppa*, *trpC*, *ureI*, and *yphC*) identified six main H. pylori groups, namely, hpAfrica1, hpAfrica2, hpEastAsia, hpAsia2, hpEurope, and hpSahul, and other more recent subpopulations; hpEastAsia was divided into the hspAmerind, hspEAsia, and hspMaori groups and hpAfrica1 into the hspWAfrica and hspSAfrica groups (4, 5).

Gastric biopsy samples were collected from the antrum of Moroccan patients during upper gastrointestinal endoscopy (6). Cultures were performed in Columbia agar base solid medium supplemented with 10% horse blood, which was made selective by the addition of Skirrow supplement (Oxoid, Basingstoke, Hampshire, United Kingdom) containing the following antibiotics: vancomycin (10 mg/liter), trimethoprim (5 mg/liter), amphotericin B (2 mg/liter), and polymyxin (2,500 IU/liter). The cultures were incubated for 6 days at 37°C in a humid and microaerobic atmosphere using microaerobic atmosphere-generating sachets (CampyGen; Oxoid). H. pylori strains were identified based on the typical appearance with Gram staining and the presence of urease, oxidase, and catalase activities. Genomic DNA was extracted using a QIAamp DNA minikit (Qiagen, Courtaboeuf, France). The DNA quality of six H. pylori isolates (HP725g, HP_751, HP_151, HP_Pws, HP_106, and G4) was assessed using a NanoVue spectrophotometer (Biochrom) and quantified using a Quantus fluorometer (Promega). DNA libraries were prepared using the Nextera XT library preparation kit, and sequencing was performed in 2 × 251 cycles using the MiSeq 600-cycle v3 reagent kit (Illumina). The paired-end reads generated by Illumina sequencing were analyzed by FastQC v0.11.8 (7), and low-quality sequences were removed by Trimmomatic v0.39 (8). The trimmed sequences were assembled *de novo* with SPAdes v3.9.1 or v3.13.1 (9) or Minia v2.0.7 (10) software. All contigs were annotated by NCBI staff using the Prokaryotic Genome Annotation Pipeline (PGAP) (https://www.ncbi.nlm.nih.gov/genomes/static/Pipeline.html) based on the best-placed reference protein set and GeneMarkS+ (11). Assembly statistics for all six strains are provided in Table 1. Default parameters were used for all software tools, unless otherwise stated

**TABLE 1:** Genome statistics and genomic features of six Moroccan H. pylori strains

Strain	GenBank accession no.	SRA accession no.	Genome size (bp)	Total no. of reads	Avg coverage (×)	Total no. of RNAs	No. of coding sequences	No. of contigs	Contig *N*_50_ (bp)	G+C content (%)
HP725g	MTLF00000000	SRR8369177	1,712,620	1,986,237	540	44	1,814	282	64,088	39.3
HP_106	MWQM00000000	SRR8369178	1,604,510	2,069,431	540	42	1,590	126	27,789	39.2
HP_Pws	MUHJ00000000	SRR8369176	1,627,601	1,978,196	580	44	1,612	101	47,942	39.3
G4	MWUG00000000	SRR8369179	1,606,340	2,083,279	540	42	1,595	122	29,595	39.2
HP_751	WTXQ00000000	SRR11886358	1,865,241	1,324,943	380	47	1,865	306	57,556	40.9
HP_151	WTXP00000000	SRR11886357	1,781,460	2,710,885	330	50	1,775	221	59,001	40.1

**FIG 1:**
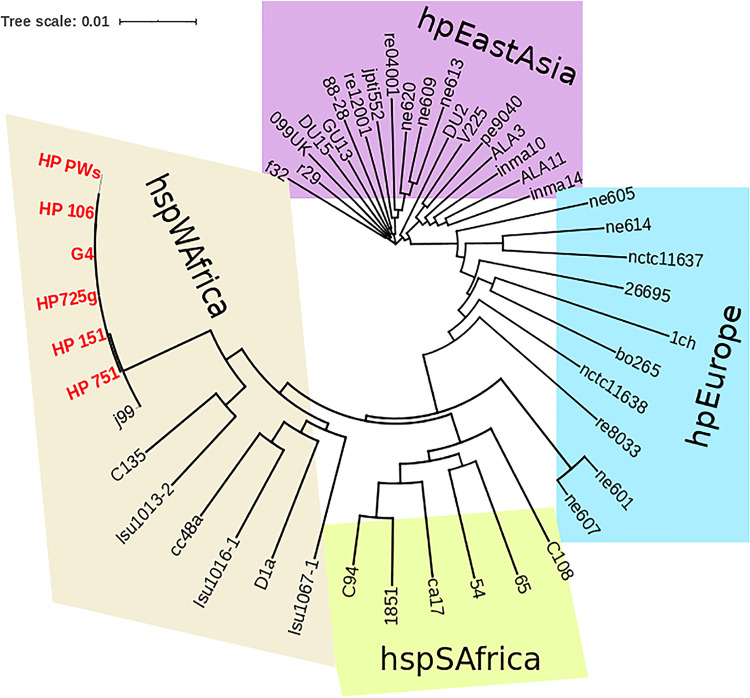
Phylogenetic analysis of six Moroccan H. pylori strains (red) and 42 worldwide strains (the name used in the phylogenetic tree represents that of the isolate recorded in the PubMLST database) by using the MLST method with seven housekeeping genes (*ureI*, *mutY*, *efp*, *ppa*, *yphC*, *atpA*, and *trpC*). A multi-FASTA file containing the concatenation of the housekeeping genes was created for each H. pylori strain. The Clustal W program in MEGA X software was used for multiple sequence alignment of the MLST genes (14). A distance-based phylogenetic tree was prepared using the maximum composite likelihood model, and bootstrap analysis was performed with 1,000 replications. A Newick tree format was generated using the neighbor-joining algorithm of MEGA X (15), and a phylogenetic tree was constructed using the Interactive Tree of Life (iTOL) platform (16). All strains were divided into four groups, i.e., hspWAfrica, hspSAfrica, hpEurope, and hpEastAsia.

For analysis of the lineage type of the six studied strains, MLST phylogeny analysis was performed based on seven standard H. pylori housekeeping genes (*ureI*, *mutY*, *efp*, *ppa*, *yphC*, *atpA*, and *trpC*). The sequences of the seven genes were extracted from whole-genome sequence data for the six H. pylori strains using the ARIBA pipeline v2.14.5 (12) and were concatenated. Besides the sequences from the Moroccan isolates, 42 H. pylori strains from different countries with a known origin were downloaded from the MLST database (http://pubmlst.org/helicobacter) (13). MLST analysis showed that the six strains belonged to the hspWAfrica group (Fig. 1). The availability of sequences for Moroccan H. pylori genomes will be beneficial for functional comparative genomic studies to greatly enhance the understanding of antibiotic resistance mechanisms occurring in this pathogen and to provide information on the genetic population lineage of Moroccan H. pylori strains.

### Data availability.

The draft genomes and the raw data for all six strains have been deposited in the DDBJ/EMBL/GenBank and DDBJ/SRA databases under accession numbers MTLF00000000, MWQM00000000, MUHJ00000000, MWUG00000000, WTXQ00000000, and WTXP00000000 (Table 1).

## References

[1] Cover TL, Blaser MJ 1996 *Helicobacter pylori* infection, a paradigm for chronic mucosal inflammation: pathogenesis and implications for eradication and prevention. Adv Intern Med 41:85–117. 8903587

[2] Cover TL, Blaser MJ 2009 *Helicobacter pylori* in health and disease. Gastroenterology 136:1863–1873. doi:10.1053/j.gastro.2009.01.073. 19457415 PMC3644425

[3] Bounder G, Boura H, SalouaNadifiyine, Jouimy MR, Bensassi M, Kadi M, Eljihad M, Badre W, Benomar H, Kettani A, Lebrazi H, Maachi F 2017 Epidemiology of *Helicobacter pylori* infection and related gastric pathologies in Moroccan population. J Life Sci 11:211–218. doi:10.17265/1934-7391/2017.05.001.

[4] Falush D, Wirth T, Linz B, Pritchard JK, Stephens M, Kidd M, Blaser MJ, Graham DY, Vacher S, Perez-Perez GI, Yamaoka Y, Mégraud F, Otto K, Reichard U, Katzowitsch E, Wang X, Achtman M, Suerbaum S 2003 Traces of human migrations in *Helicobacter pylori* populations. Science 299:1582–1585. doi:10.1126/science.1080857. 12624269

[5] Moodley Y, Linz B 2009 *Helicobacter pylori* sequences reflect past human migrations, p 62–74. *In* de Reuse H, Bereswill S (ed), Microbial pathogenomics, vol 6 Karger, Basel, Switzerland. 10.1159/000235763 19696494

[6] Bouihat N, Burucoa C, Benkirane A, Seddik H, Sentissi S, Al Bouzidi A, Elouennas M, Benouda A 2017 *Helicobacter pylori* primary antibiotic resistance in 2015 in Morocco: a phenotypic and genotypic prospective and multicenter study. Microb Drug Resist 23:727–732. doi:10.1089/mdr.2016.0264. 27996373

[7] Andrews S 2010 FastQC: a quality control tool for high throughput sequence data. https://www.bioinformatics.babraham.ac.uk/projects/fastqc.

[8] Bolger AM, Lohse M, Usadel B 2014 Trimmomatic: a flexible trimmer for Illumina sequence data. Bioinformatics 30:2114–2120. doi:10.1093/bioinformatics/btu170. 24695404 PMC4103590

[9] Bankevich A, Nurk S, Antipov D, Gurevich AA, Dvorkin M, Kulikov AS, Lesin VM, Nikolenko SI, Pham S, Prjibelski AD, Pyshkin AV, Sirotkin AV, Vyahhi N, Tesler G, Alekseyev MA, Pevzner PA 2012 SPAdes: a new genome assembly algorithm and its applications to single-cell sequencing. J Comput Biol 19:455–477. doi:10.1089/cmb.2012.0021. 22506599 PMC3342519

[10] Chikhi R, Rizk G 2013 Space-efficient and exact de Bruijn graph representation based on a Bloom filter. Algorithms Mol Biol 8:22. doi:10.1186/1748-7188-8-22. 24040893 PMC3848682

[11] Tatusova T, DiCuccio M, Badretdin A, Chetvernin V, Nawrocki EP, Zaslavsky L, Lomsadze A, Pruitt KD, Borodovsky M, Ostell J 2016 NCBI Prokaryotic Genome Annotation Pipeline. Nucleic Acids Res 44:6614–6624. doi:10.1093/nar/gkw569. 27342282 PMC5001611

[12] Hunt M, Mather AE, Sánchez-Busó L, Page AJ, Parkhill J, Keane JA, Harris SR 2017 ARIBA: rapid antimicrobial resistance genotyping directly from sequencing reads. Microb Genom 3:e000131. doi:10.1099/mgen.0.000131. 29177089 PMC5695208

[13] Jolley KA, Bray JE, Maiden MCJ 2018 Open-access bacterial population genomics: BIGSdb software, the PubMLST.org website and their applications. Wellcome Open Res 3:124. doi:10.12688/wellcomeopenres.14826.1. 30345391 PMC6192448

[14] Kumar S, Stecher G, Li M, Knyaz C, Tamura K 2018 MEGA X: molecular evolutionary genetics analysis across computing platforms. Mol Biol Evol 35:1547–1549. doi:10.1093/molbev/msy096. 29722887 PMC5967553

[15] Tamura K, Nei M, Kumar S 2004 Prospects for inferring very large phylogenies by using the neighbor-joining method. Proc Natl Acad Sci U S A 101:11030–11035. doi:10.1073/pnas.0404206101. 15258291 PMC491989

[16] Letunic I, Bork P 2019 Interactive Tree of Life (iTOL) v4: recent updates and new developments. Nucleic Acids Res 47:W256–W259. doi:10.1093/nar/gkz239. 30931475 PMC6602468

